# miR‐212 downregulation contributes to the protective effect of exercise against non‐alcoholic fatty liver *via* targeting FGF‐21

**DOI:** 10.1111/jcmm.12733

**Published:** 2015-12-09

**Authors:** Junjie Xiao, Yihua Bei, Jingqi Liu, Jasmina Dimitrova‐Shumkovska, Dapeng Kuang, Qiulian Zhou, Jin Li, Yanning Yang, Yang Xiang, Fei Wang, Changqing Yang, Wenzhuo Yang

**Affiliations:** ^1^ Regeneration and Ageing LabExperimental Center of Life SciencesSchool of Life ScienceShanghai UniversityShanghaiChina; ^2^ Shanghai Key Laboratory of Bio‐Energy CropsSchool of Life SciencesShanghai UniversityShanghaiChina; ^3^ Division of Gastroenterology and HepatologyDigestive Disease InstituteTongji HospitalTongji University School of MedicineShanghaiChina; ^4^ Department of Experimental Biochemistry and PhysiologyFaculty of Natural Sciences and MathematicsUniversity Ss Cyril and MethodiusSkopjeRepublic of Macedonia; ^5^ State Key Laboratory of Pharmaceutical Biotechnology and Department of BiochemistryNanjing UniversityNanjingChina

**Keywords:** exercise, miR‐212, FGF‐21, NAFLD, steatosis

## Abstract

Non‐alcoholic fatty liver disease (NAFLD) is associated with obesity and lifestyle, while exercise is beneficial for NAFLD. Dysregulated microRNAs (miRs) control the pathogenesis of NAFLD. However, whether exercise could prevent NAFLD *via* targeting microRNA is unknown. In this study, normal or high‐fat diet (HF) mice were either subjected to a 16‐week running program or kept sedentary. Exercise attenuated liver steatosis in HF mice. MicroRNA array and qRT‐PCR demonstrated that miR‐212 was overexpressed in HF liver, while reduced by exercise. Next, we investigated the role of miR‐212 in lipogenesis using HepG2 cells with/without long‐chain fatty acid treatment (±FFA). FFA increased miR‐212 in HepG2 cells. Moreover, miR‐212 promoted lipogenesis in HepG2 cells (±FFA). Fibroblast growth factor (FGF)‐21, a key regulator for lipid metabolism, was negatively regulated by miR‐212 at protein level in HepG2 cells. Meanwhile, FFA downregulated FGF‐21 both at mRNA and protein levels in HepG2 cells. Also, FGF‐21 protein level was reduced in HF liver, while reversed by exercise *in vivo*. Furthermore, siRNA‐FGF‐21 abolished the lipogenesis‐reducing effect of miR‐212 inhibitor in HepG2 cells (±FFA), validating FGF‐21 as a target gene of miR‐212. These data link the benefit of exercise and miR‐212 downregulation in preventing NAFLD *via* targeting FGF‐21.

## Introduction

Non‐alcoholic fatty liver disease (NAFLD) is an important public health problem closely associated with genetic factors, obesity, type 2 diabetes and lifestyle act 1. Non‐alcoholic fatty liver disease comprises a spectrum of hepatic disorders, usually beginning with simple steatosis, characterized by accumulation of triglyceride (TG) within lipid droplets in hepatocytes, which can possibly progress to non‐alcoholic steatohepatitis, liver cirrhosis, and even worse, hepatocellular carcinoma 2. Although the dysregulated metabolism of TG and insulin resistance have been known to be critical for the development of NAFLD, the pathogenesis of NAFLD is incompletely understood and current therapeutic strategies are far from satisfactory 3.

Population‐based studies indicate that NAFLD is associated with sedentary life style 4. Physical exercise has been known to be beneficial for NAFLD prevention and treatment 5, 6, 7, 8. It has been reported that physical exercise at least once a week was effective to attenuate hepatic steatosis in NAFLD patients 9. Physical exercise is supposed to reduce intrahepatic TG accumulation, improve insulin sensitivity and attenuate oxidative stress 10. To those who have cardiorespiratory limitations, resistance exercise also displays beneficial effects on NAFLD through improving insulin sensitivity, reducing intrahepatic lipids and inducing hepatic fat oxidation 11, 12, 13. A better understanding of the molecular mechanisms mediating the protective effect of exercise against NAFLD is of great importance.

MicroRNAs (miRNAs, miRs) are central post‐transcriptional negative regulators of gene expression through inducing mRNA degradation and/or translational inhibition 14, 15. Recent studies reveal the emerging roles of miRNA deregulation in the development of NAFLD, such as miR‐122, ‐34a, ‐155, ‐10b, ‐467b, 216, ‐302a and ‐33a/b, *via* targeting fatty acid synthase (FAS), acetyl‐CoA carboxylase 1 and 2 (ACC1/ACC2), sterol regulatory element binding protein‐1c (SREBP‐1c), sirtuin‐1 (SIRT‐1) and ATP‐binding cassette‐A1 transporter 16, 17. More recently, miR‐29 inhibition has been reported to reduce lipogenic programs *via* targeting SIRT‐1 and aryl hydrocarbon receptor 18. Additionally, dysregulated circulating miRNAs have also been detected in NAFLD patients, including miR‐122 and miR‐192 19. Besides of their biological roles in fundamental cellular processes (proliferation, apoptosis, migration and differentiation), miRNAs also contribute to the control of hepatic metabolic functions, oxidative stress, inflammation and insulin resistance that are considered as key factors involved in the pathogenesis of NAFLD 20, 21, 22, 23, 24, 25. Interestingly, exercise has been reported to exert beneficial effects in the prevention and treatment of obesity, diabetes and cardiovascular diseases by the regulation of miRNA biology 26, 27, 28, 29, 30, 31, 32. However, the potential of miRNA in mediating the protective effect of exercise against NAFLD remains largely unknown.

In the present study, we demonstrate that hepatic miR‐212 is upregulated in high‐fat (HF)‐diet fed mice, while exercise protects the liver from HF‐diet induced hepatic steatosis with blunted miR‐212 expression. Our data further show that miR‐212 participates in the lipogenesis in HepG2 cells *in vitro*, through targeting fibroblast growth factor (FGF)‐21 which is a central regulator for lipid metabolism. This work provides strong evidence suggesting that miR‐212 might be a novel therapeutic target mimicking the benefit of exercise in the treatment of NAFLD.

## Materials and methods

### Animal experimentation

Male C57BL/6 mice aged 8–10 weeks were purchased from SLAC Laboratory Animal Center, Shanghai. Mice were housed in a temperature‐controlled room on a 12 hrs light/dark cycle and fed *ad libitum* with a HF or control diet for 16 weeks. Mice were randomized into four groups: (*i*) control group (*n* = 10), mice fed with standard chow (*n* = 10); (*ii*) exercise group (*n* = 10), mice fed with standard chow and subjected to exercise; (*iii*) HF diet group (*n* = 10), mice fed with HF diet (20.26% carbohydrate, 19.74% protein, and 60% fat) and (*iv*) HF diet & exercise group (*n* = 10). Exercised‐mice were put on a running machine at the speed 10 m/min. for 60 min./day for a period of 16 weeks. Bodyweight was recorded once a week during the study. After 16 weeks, animals were weighted and killed. Lee's index was calculated as bodyweight (g)^(1/3) ×1000/naso‐anal length (cm) to evaluate obesity degree. The study protocol was reviewed and approved by the ethics committee of Shanghai University and all animal experiments were conducted under the guidelines on humane use and care of laboratory animals for biomedical research published by National Institutes of Health (No. 85‐23, revised 1996).

### Histopathological analysis

Liver segments (three selected specimens from different regions of the liver) were removed from each mouse, fixed in 4% buffered formalin and processed for embedding in paraffin. The 5 μm‐thick liver sections were stained with haematoxylin and eosin and Oil Red O staining for evaluation of hepatic steatosis.

### Serum analysis

Serum alanine transaminase (ALT) and aspartate transaminase (AST) activities (U/l), as well as total cholesterol (TCH) and TG levels (mmol/l) were measured using routine clinical chemical assays (Nanjing Jiancheng, China).

### Microarray analysis

Total RNA were isolated from liver tissues and quantified by the NanoDrop ND‐2100 (Thermo Scientific, Hudson, NH, USA) and the RNA integrity was assessed using Agilent 2100 (Agilent Technologies, Palo Alto, CA, USA). The sample labelling, microarray hybridization and washing were performed based on the manufacturer's standard protocols. Briefly, total RNA were tailed with Poly A and then labelled with Biotin. After, the labelled RNA were hybridized for 16 hrs at 48°C on Affemetrix miRNA 3.0 Array. GeneChips were washed and stained in the Affymetrix Fluidics Station 450. The arrays were scanned by the Affymetrix Scanner 3000 (Affymetrix, Cleveland, OH, USA). Affymetrix GeneChip Command Console software (version 4.0; Affymetrix) was used to analyse array images to get raw data and then offered Robust Multi‐Array Analysis (RMA) normalization. Next, Genespring software (version 12.5; Agilent Technologies) was used to proceed the data analysis. Probes that at least 75 percent of samples in any 1 condition out of 2 conditions have flags in ‘Present’ were chosen for further data analysis. Differentially expressed miRNAs were then identified through fold change as well as *P*‐value calculated using *t*‐test. The threshold set for up‐ and down‐regulated genes was a fold change ≥2.0 and a *P* ≤ 0.05. The MIAME‐compliant data have been submitted to Gene Expression Omnibus (platform ID: GSE65978).

### Cell culture and FFA treatment

Human hepatocellular carcinoma HepG2 cells were purchased from the Cell Bank of Chinese Academy of Sciences (Shanghai, China). Cells were cultured in high glucose‐DMEM (Hyclone, Logan, UT, USA) containing 10% foetal bovine serum (Hyclone) and 1% penicillin/streptomycin in a humidified atmosphere of 5% CO_2_ at 37°C. HepG2 cells were treated with 1 mM long‐chain fatty acid (FFA) (oleate: palmitate at a molar ration of 2:1; Sigma‐Aldrich, St. Louis, MO, USA) in 1% bovine serum albumin for 24 hrs to induce lipogenesis *in vitro*.

### Cell transfection

HepG2 cells were transfected with miR‐212 mimics (50 nM), miR‐212 inhibitor (100 nM), small interfering RNA (siRNA)‐FGF21 (75 nM), and their negative controls (NC; RiboBio, Guangzhou China) for 48 hrs using Lipofectamine 2000 (Invitrogen, Carlsbad, CA, USA) according to the manufacturer's instructions. The efficiency of transfection was evaluated by real‐time PCR.

### Nile Red staining for intracellular lipid droplets analysis

HepG2 cells were seeded at 1.5 × 10^5^ cells/well in 24‐well plates. After treated with 1 mM FFA for 24 hrs, cells were washed twice with PBS and fixed with 4% paraformaldehyde for 15 min. Intracellular lipids were stained with Nile Red dye (0.1 μmol/ml) for 15 min. Cell nuclei were stained with Hoescht 33258 dye for 20 min. The staining process was conducted at room temperature avoiding direct light. Images were acquired with a fluorescence microscope with magnification of 100× and 200× (Leica, Wetzlar, Germany).

### Flow cytofluorometry for intracellular lipid content assessment

Intracellular lipid content was determined by flow cytofluorometry based on Nile Red staining. Cells were washed twice with PBS and detached by trypsinization. After centrifugation at 166***g*** for 5 min., the cell pellet was resuspended in 1 ml PBS and incubated with 0.75 μg/ml Nile Red dye for 15 min. at room temperature. Nile Red intracellular fluorescence was determined by flow cytofluorometry using MoFlo XDP Cell Sorter (Beckman Coulter, Miami, FL, USA) on the FL3 emission channel through a 585 ± 21 nm band pass filter, following excitation with an argon ion laser source at 488 nm 33. A total of 15,000 cells were collected and data were analysed using FlowJo software (Treestar Inc., Ashland, OR, USA). Fluorescence intensity was expressed indirectly as the percentage of events above the median value of fluorescence.

### Real time quantitative PCR

Isolation of total RNA was performed using Trizol reagent (Invitrogen, Paisley, UK). For mRNA analysis, a total of 400 ng RNA was used for cDNA synthesis using Bio‐Rad iScripTM cDNA Synthesis Kit (Bio‐Rad, Richmond, CA, USA). PCR amplification was carried out with Takara SYBR Premix Ex Taq^™^ (Tli RNaseH Plus, Takara, Kusatsu, Japan) in CFX96TM Real‐Time PCR Detection System (Bio‐Rad). β‐actin was used as internal control. For miRNA analysis, the Bulge‐LoopTM miRNA qPCR Primer Set (RiboBio) was used to detect miR‐212 expression by qRT‐PCR with Takara SYBR Premix Ex Taq^™^ in CFX96TM Real‐Time PCR Detection System. U6 and 5s were used as internal controls for *in vivo* and *in vitro* experiments, respectively. The utilized primers were listed in Table 1.

**Table 1:** List of utilized primers for qRT‐PCR

Gene	Species	Forward primer	Reverse primer
FGF‐21	Mouse	5′‐CTGCTGGGGGTCTACCAAG‐3′	5′‐CTGCGCCTACCACTGTTCC‐3′
β‐actin	Mouse	5′‐GGCTGTATTCCCCTCCATCG‐3′	5′‐CCAGTTGGTAACAATGCCATGT‐3′
FGF‐21	Human	5′‐ATGGATCGCTCCACTTTGACC‐3′	5′‐GGGCTTCGGACTGGTAAACAT‐3′
β‐actin	Human	5′‐CATGTACGTTGCTATCCAGGC‐3′	5′‐CTCCTTAATGTCACGCACGAT‐3′

### Western blot analysis

Liver tissues or HepG2 cells were lysed using RIPA lysis buffer (Beyotime Institute of Biotechnology, China) containing 1% phenylmethanesulfonyl fluoride. A total of 30 μg protein was subjected to electrophoreses on 12% SDS‐Page gels, transferred to Polyvinylidene fluoride membrane (PVDF) membranes, and incubated overnight at 4°C with primary antibody of FGF21 (1:500 dilution, ST1615; Merck Millipore, Darmstadt, Germany). After incubation with corresponding HRP‐conjugated secondary antibody, protein bands were visualized using enhanced chemiluminescence system (Pierce Biotechnology Inc., Rockford, IL, USA). Densitometric analysis of protein bands was performed using Image Lab software (Bio‐Rad). β‐actin (1:10,000 dilution, 1854‐1; Huaan, Hangzhou, Zhejiang, China) was used as loading control.

### Statistical analysis

The relative expression levels of mRNA and miRNA were calculated using the 2^−ΔΔCt^ method. Data are expressed as mean ± S.E.M. One‐way anova test was performed to analyse difference between groups, followed by Bonferroni's post‐hoc test for multiple comparisons. All analysis were performed using (SPSS software, International Business Machines Corporation, Armonk, NY, USA) (version 19.0). *P*‐value less than 0.05 was considered significant.

## Results

### Exercise reduces HF‐diet induced liver steatosis and hepatic injury

**Figure 1:**
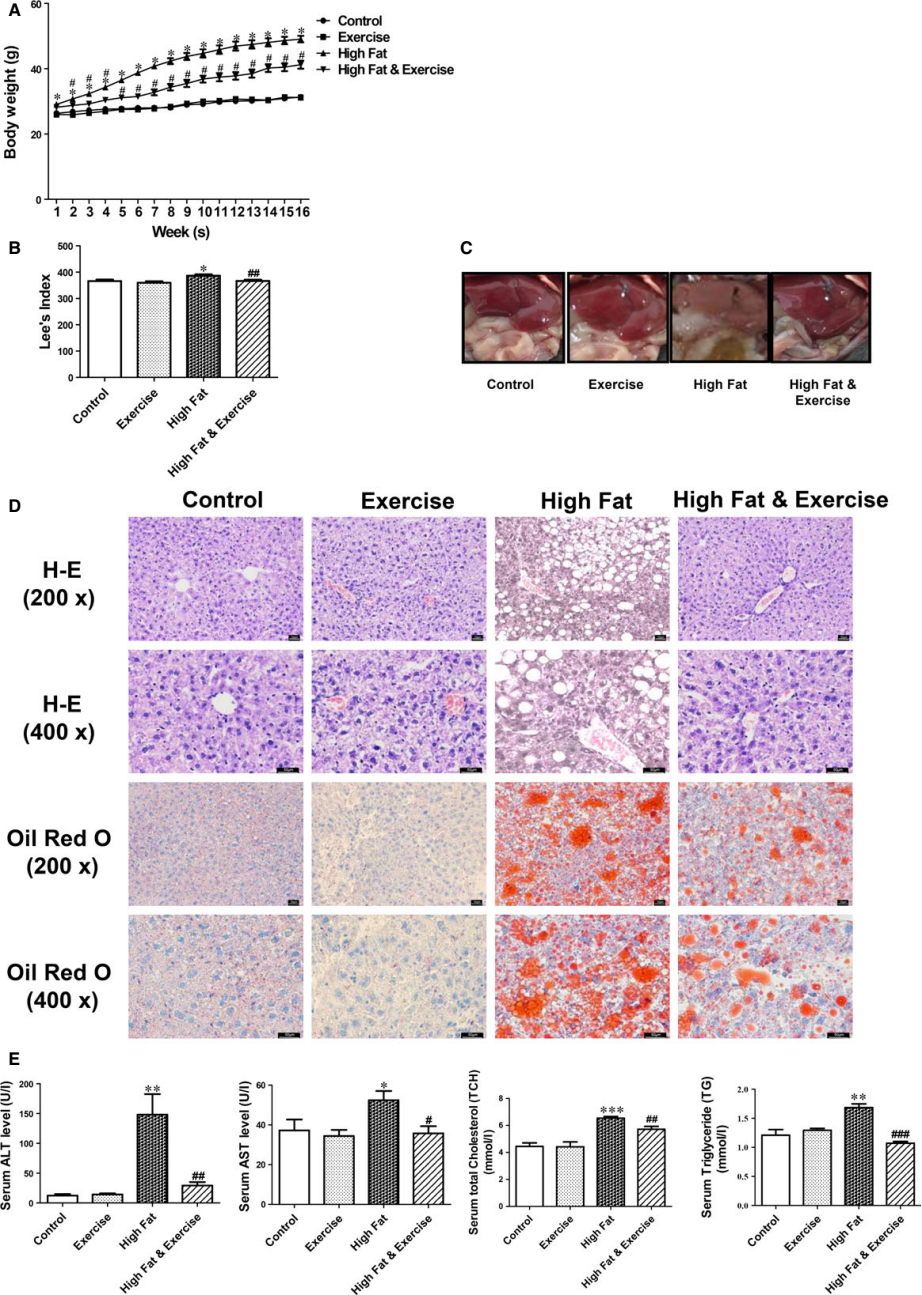
Effects of exercise on liver steatosis and hepatic injury. (**A**) The bodyweight of C57BL/6 mice in control group, exercise group, high fat (HF)‐diet group, and HF‐diet & exercise group. (**B**) Lee's index was used to evaluate the degree of obesity, which was significantly higher in HF‐diet group compared to control group, whereas HF‐diet induced increase in Lee's index was reversed by exercise. (**C**) Macroscopic findings and (**D**) representative images of haematoxylin and eosin staining and Oil Red O staining of the liver demonstrated reduced liver steatosis in exercised HF mice compared to sedentary HF mice. (**E**) Serum ALT, AST, TCH, and TG levels were increased in HF mice, while decreased in exercised HF mice. Results represented as mean ± S.E.M. (*n* = 10). *P < 0.05, **P < 0.01, ***P < 0.001 *versus* control group; #*P* < 0.05, ##*P* < 0.01, ###*P* < 0.001 *versus* HF‐diet group; scale bar = 50 μm.

After a 16‐week HF‐diet, mice became markedly obese compared to controls, while HF‐diet induced increase in bodyweight and Lee's index was reduced by exercise (Fig. 1A and B). The livers of HF group were macroscopically enlarged and pale in colour (Fig. 1C). Haematoxylin and eosin staining and Oil Red O staining for liver tissues showed increased accumulation of lipid droplets in hepatocytes (Fig. 1D). Meanwhile, HF mice displayed elevated serum levels of ALT, AST, TCH and TG (Fig. 1E). All of these changes were significantly attenuated by exercise (Fig. 1C–E), indicative of protective effect of exercise against liver steatosis and hepatic injury in HF mice.

### Exercise diminishes upregulation of miR‐212 in livers of HF mice

**Figure 2:**
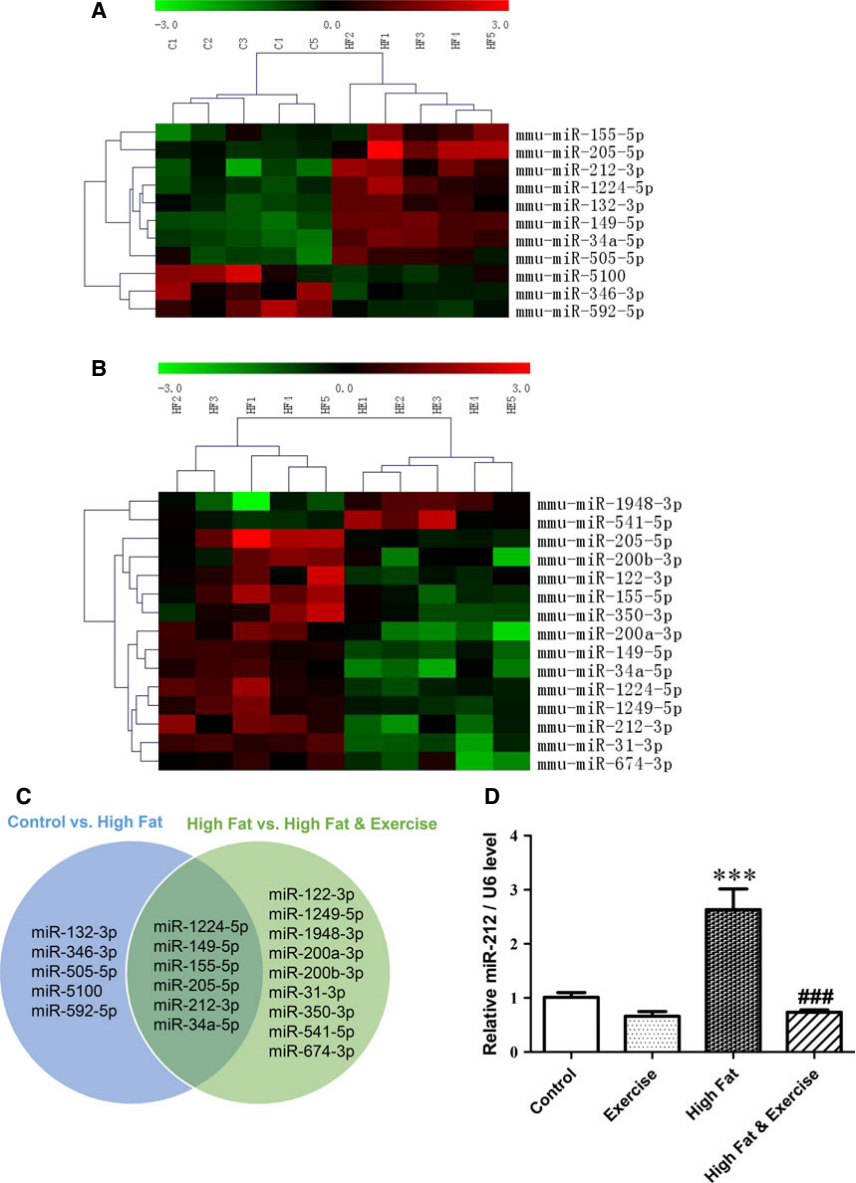
High fat (HF) diet induced upregulation of miRNA‐212 is blunted by exercise. (**A**) Heat map demonstrated the dysregulated microRNAs in HF‐diet group (HF,* n* = 5) *versus* control group (C, *n* = 5). (**B**) Heat map demonstrated the dysregulated microRNAs in HF‐diet & Exercise group (HE,* n* = 5) *versus* HF‐diet group (HF,* n* = 5). (**C**) Schematic diagram showed that six miRNAs, including miR‐34a, ‐149, ‐155, ‐205, ‐212 and ‐1224, were found to be upregulated in HF‐diet group *versus* control group, while downregulated in HF‐diet & exercise group *versus* HF‐diet group. (**D**) miR‐212 expression was confirmed by qRT‐PCR (*n* = 5). Results represented as mean ± S.E.M. ***P < 0.001 *versus* control group; ###*P* < 0.001 *versus* HF‐diet group.

To determine the profiles of hepatic miRNA in HF mice with or without exercise compared to normal‐diet mice, microarray analysis was performed using Affymetrix 3.0. Eleven miRNAs (miR‐1224‐5p, ‐132‐3p, ‐149‐5p, ‐155‐5p, ‐205‐5p, ‐212‐3p, ‐346‐3p, ‐34a‐5p,‐505‐5p, ‐5100 and ‐592‐5p) were found to be dysregulated in HF mice *versus* control mice (Table 2 and Fig. 2A). Meanwhile, 15 miRNAs (miR‐122‐3p, ‐1224‐5p, ‐1249‐5p, ‐149‐5p, ‐155‐5p, ‐1948‐3p, ‐200a‐3p, ‐200b‐3p, ‐205‐5p, ‐212‐3p, ‐31‐3p, ‐34a‐5p, ‐350‐3p, ‐541‐5p, ‐674‐3p) were found to be dysregulated in exercised HF mice *versus* sedentary HF mice (Table 3 and Fig. 2B). Interestingly, the upregulation of hepatic miR‐34a, ‐149, ‐155, ‐205, ‐212 and ‐1224 in HF mice was downregulated by exercise (Fig. 2C). Among these miRNA, miR‐205 and miR‐212 were the most upregulated miRNA in livers of HF mice *versus* control mice (FC (abs) = 5.011 and 4.361, respectively; Table 2). Next, by searching in miRWalk database, we found that FGF‐21, a central regulator for lipid metabolism, is a previously validated target gene of miR‐212, while no validated target gene of miR‐205 has been found to be involved in NAFLD. Thus, the current study was focused on miR‐212. By using qRT‐PCR, we further verified that hepatic miR‐212 level was induced by HF‐diet, while diminished by exercise in HF mice (Fig. 2D). These results prompted us to further investigate the role of miR‐212 dysregulation in the development of liver steatosis and hepatic injury.

**Table 2:** Dysregulated microRNAs in high fat‐diet group *versus* control group

Systematic name	*P*‐value	Fold‐change	Regulation
mmu‐miR‐1224‐5p	0.001	2.905	Up
mmu‐miR‐132‐3p	0.001	2.445	Up
mmu‐miR‐149‐5p	0.000	4.207	Up
mmu‐miR‐155‐5p	0.029	2.453	Up
mmu‐miR‐205‐5p	0.007	5.011	Up
mmu‐miR‐212‐3p	0.001	4.361	Up
mmu‐miR‐346‐3p	0.013	2.400	Down
mmu‐miR‐34a‐5p	0.000	3.893	Up
mmu‐miR‐505‐5p	0.010	2.358	Up
mmu‐miR‐5100	0.031	2.772	Down
mmu‐miR‐592‐5p	0.004	2.670	Down

**Table 3:** Dysregulated miRNAs in high fat‐diet & exercise group *versus* high fat‐diet group

Systematic name	*P*‐value	Fold‐change	Regulation
mmu‐miR‐122‐3p	0.037	2.296	Down
mmu‐miR‐1224‐5p	0.003	2.539	Down
mmu‐miR‐1249‐5p	0.002	2.151	Down
mmu‐miR‐149‐5p	0.000	2.375	Down
mmu‐miR‐155‐5p	0.008	2.922	Down
mmu‐miR‐1948‐3p	0.013	3.291	Up
mmu‐miR‐200a‐3p	0.002	4.125	Down
mmu‐miR‐200b‐3p	0.048	2.700	Down
mmu‐miR‐205‐5p	0.012	4.273	Down
mmu‐miR‐212‐3p	0.004	3.421	Down
mmu‐miR‐31‐3p	0.000	3.298	Down
mmu‐miR‐34a‐5p	0.001	3.224	Down
mmu‐miR‐350‐3p	0.047	2.430	Down
mmu‐miR‐541‐5p	0.024	2.549	Up
mmu‐miR‐674‐3p	0.027	2.344	Down

### miR‐212 is induced in FFA‐treated HepG2 cells *in vitro*

**Figure 3:**
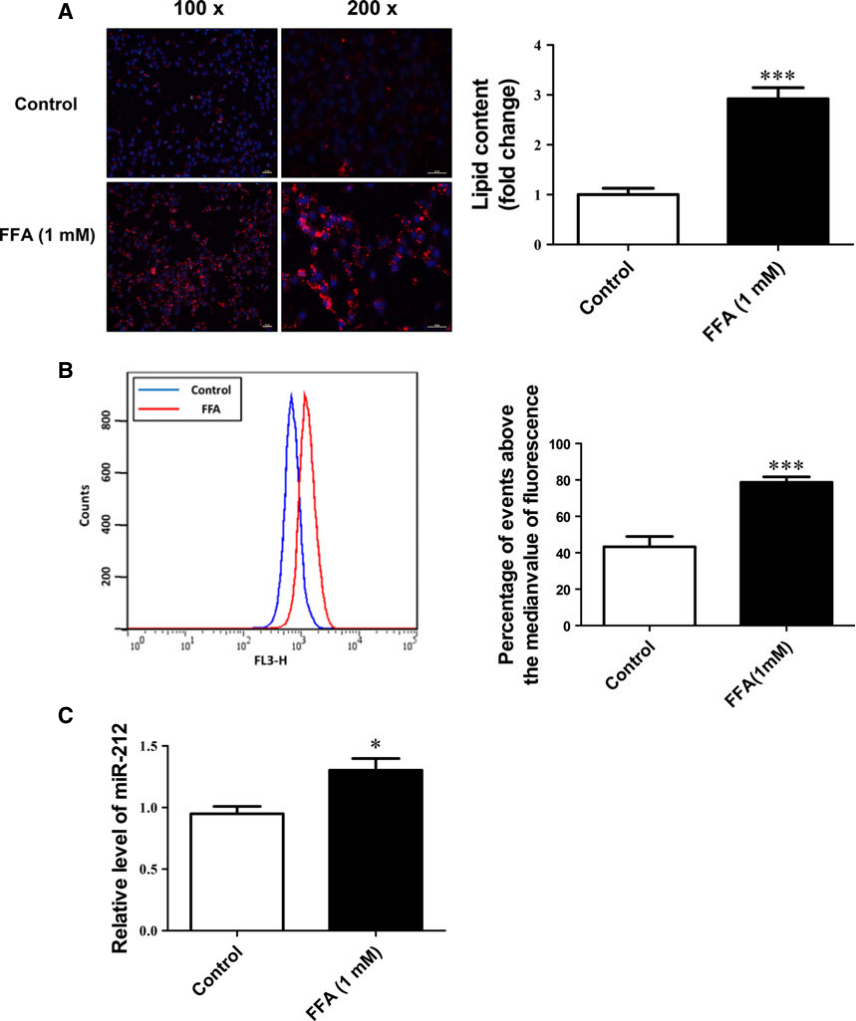
Upregulation of miR‐212 in FFA‐treated HepG2 cells *in vitro*. (**A**) Nile Red staining and (**B**) flow cytofluorometry showed increased lipid content in FFA‐treated HepG2 cells (*n* = 4 and *n* = 5, respectively). (**C**) The level of miR‐212 was induced in FFA‐treated HepG2 cells as determined by qRT‐PCR (*n* = 6). Results represented as mean ± S.E.M. *P < 0.05, ***P < 0.001 *versus* control group; scale bar=50 μm.

HepG2 cells with or without FFA treatment were used to examine the role of miR‐212 in lipogenesis *in vitro*. FFA‐treated HepG2 cells demonstrated increased intracellular lipid content as represented by Nile Red staining (Fig. 3A) and Nile Red intracellular fluorescence determined by flow cytofluorometry (Fig. 3B). Moreover, FFA‐treated HepG2 cells displayed upregulation of miR‐212 level (Fig. 3C), indicating that miR‐212 could also be induced in an *in vitro* cell model mimicking NAFLD.

### miR‐212 participates in lipogenesis in HepG2 cells regardless of FFA treatment

**Figure 4:**
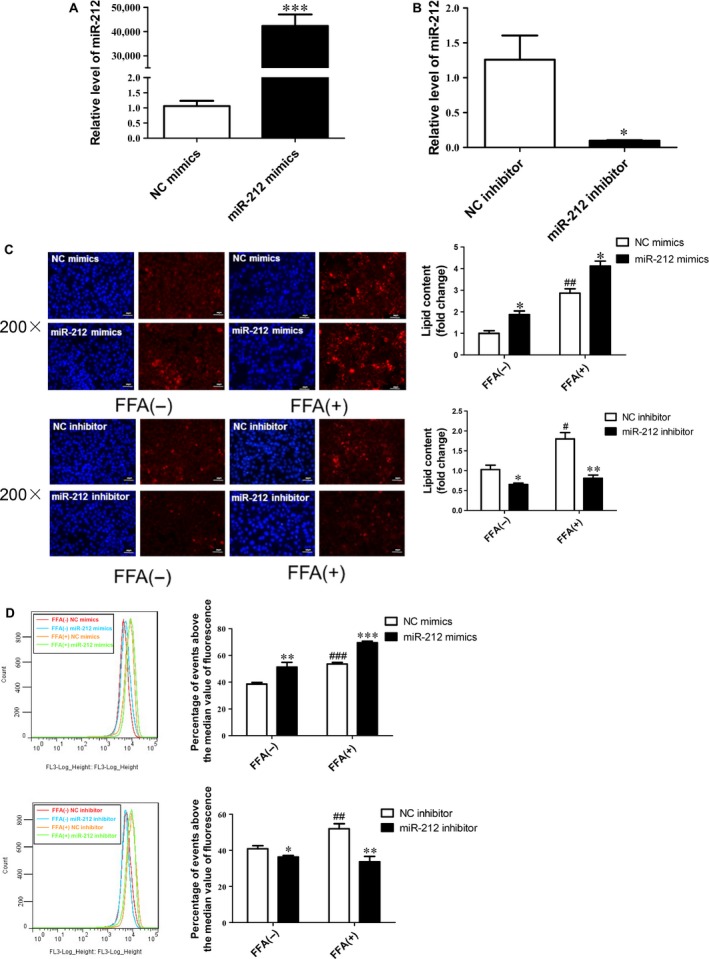
Effects of miR‐212 on lipogenesis in HepG2 cells. The effects of miR‐212 mimic (**A**) and miR‐212 inhibitor (**B**) transfection were validated by qRT‐PCR (*n* = 6). (**C**) Nile Red staining and (**D**) flow cytofluorometry demonstrated that miR‐212 mimics increased, while miR‐212 inhibitor decreased lipid content in HepG2 cells regardless of FFA treatment (*n* = 4 and *n* = 5, respectively). Results represented as mean ± S.E.M. *P < 0.05, **P < 0.01, ***P < 0.001 *versus* negative control (NC); #*P* < 0.05, ##*P* < 0.01, ###*P* < 0.001 *versus* NC in HepG2 cells in the absence of FFA treatment; scale bar=50 μm.

To determine whether miR‐212 could regulate lipogenesis *in vitro*, HepG2 cells were transfected with miR‐212 mimics, inhibitor or their NC for 48 hrs. As measured by qRT‐PCR, miR‐212 mimics upregulated, while miR‐212 inhibitor downregulated miR‐212 level in HepG2 cells (Fig. 4A and B), confirming that the inhibitors and mimics used in this study took effects. Next, we demonstrated that miR‐212 mimics increased, while miR‐212 inhibitor reduced intracellular lipid content in HepG2 cells regardless of FFA treatment using Nile Red staining and flow cytofluorometry (Fig. 4C and D). These data provide important evidence showing that miR‐212 might be a crucial regulator for lipogenesis.

### FGF‐21 is a target gene of miR‐212 involved in lipogenesis

**Figure 5:**
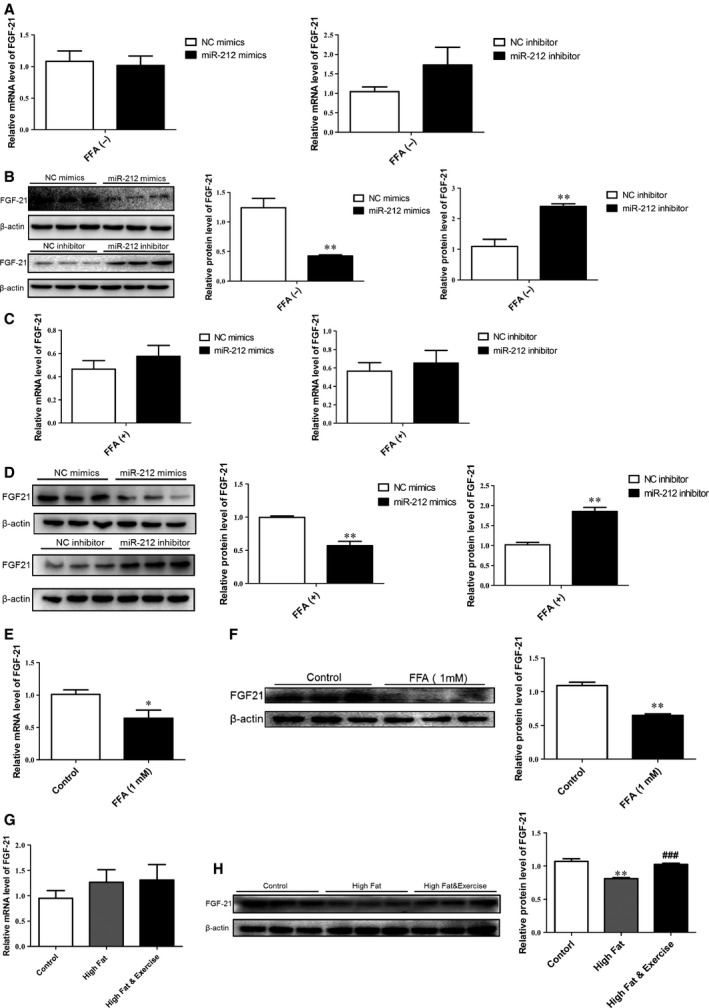
FGF‐21 is negatively regulated by miR‐212. mRNA level of FGF‐21 remained unchanged by miR‐212 mimics or inhibitor transfection in HepG2 cells without FFA (**A**) or with FFA (**C**) treatment (*n* = 6). miR‐212 negatively regulated FGF‐21 at protein level in HepG2 cells without FFA (**B**) or with FFA (**D**) treatment (*n* = 3). FFA treatment caused downregulation of FGF‐21 at both mRNA (**E**) and protein level (**F**) in HepG2 cells (*n* = 6 and *n* = 3, respectively). (**G**) FGF‐21 was not changed at mRNA level among control group, HF‐diet group, and HF‐diet & exercise group (*n* = 6). (**H**) FGF‐21 protein level was reduced in H‐F diet group, while restored in H‐F diet & exercise group (*n* = 3). Results represented as mean ± S.E.M. *P < 0.05, **P < 0.01 *versus* control; ###*P* < 0.001 *versus* HF‐diet group.

As shown in miRWalk database, FGF‐21 is a validated target gene of miR‐212. Knowing that FGF‐21 is a central regulator contributing to lipid metabolism, we continued to examine whether FGF‐21 could be a target gene of miR‐212 involved in lipogenesis. We found that miR‐212 mimics repressed, while miR‐212 inhibitor induced the expression of FGF‐21 at protein but not mRNA level in HepG2 cells regardless of FFA treatment (Fig. 5A–D). Meanwhile, FFA‐treated HepG2 cells also displayed a reduction in FGF‐21 expression at both mRNA and protein level (Fig. 5E and F). Furthermore, we tested FGF‐21 expression in mice liver samples showing that the protein level but not mRNA level of FGF‐21 was reduced by HF‐diet while reversed with exercise in HF mice (Fig. 5G and H). These data reveal that FGF‐21 could be negatively regulated by miR‐212 and/or under conditions of lipogenesis.

**Figure 6:**
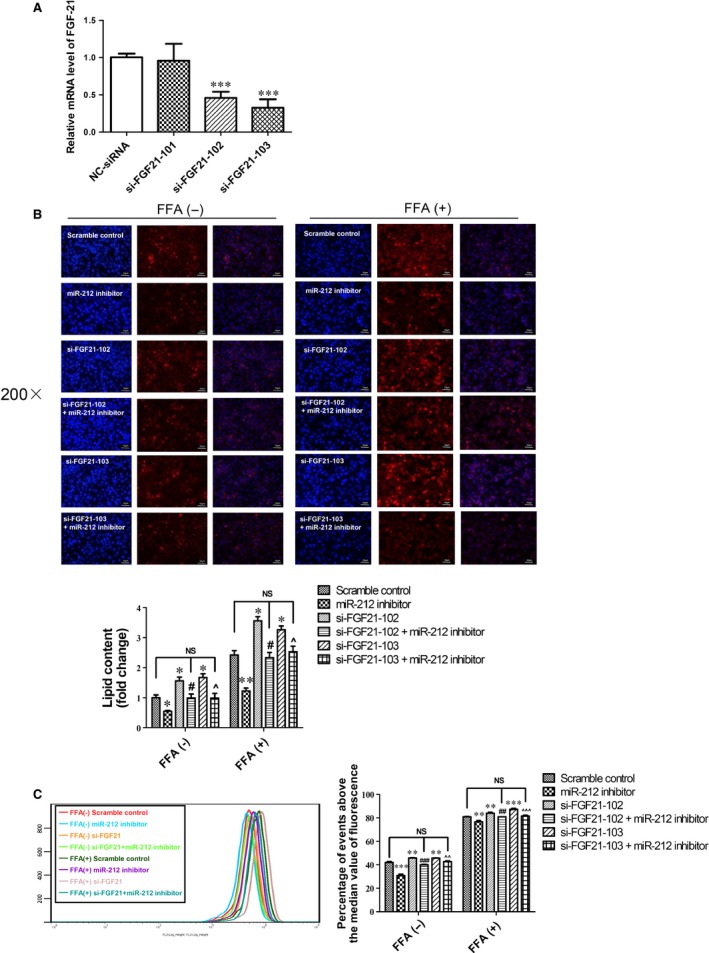
FGF‐21 is a target gene of miR‐212 involved in lipogenesis. (**A**) The inhibitory effect of three different si‐FGF‐21 was determined by qRT‐PCR (*n* = 6). (**B**) Nile Red staining and (**C**) flow cytofluorometry demonstrated that interfering FGF‐21 significantly abolished the inhibitory effect of miR‐212 inhibitor on lipogenesis in HepG2 cells regardless of FFA treatment (*n* = 4 and *n* = 5, respectively). Results represented as mean ± S.E.M. *P < 0.05, **P < 0.01, ***P < 0.001 *versus* Scramble control #*P* < 0.05 *versus* si‐FGF‐21‐102 group; ^*P* < 0.05 *versus* si‐FGF‐21‐103 group; scale bar=50 μm.

Next, FGF‐21 expression was silenced using si‐FGF‐21 which aimed to clarify if FGF‐21 was responsible for the effects of miR‐212 in lipogenesis. FGF‐21 mRNA level was efficiently suppressed by transfection of HepG2 cells with si‐FGF‐21‐102 and si‐FGF‐21‐103 (Fig. 6A). As examined by Nile Red staining and flow cytofluorometry, silencing FGF‐21 (by either si‐FGF‐21‐102 or si‐FGF‐21‐103) caused notable increase in lipid content in HepG2 cells regardless of FFA treatment (Fig. 6B and C). Furthermore, silencing FGF‐21 significantly blunted the inhibitory effect of miR‐212 inhibitor on lipogenesis in HepG2 cells (Fig. 6B and C). These data clearly indicate that FGF‐21 is a target gene of miR‐212 involved in lipogenesis.

## Discussion

Exercise is known to be beneficial for NAFLD treatment. Recent studies have shown the critical involvement of miRNA in NAFLD. However, it is unclear whether exercise could prevent NAFLD *via* miRNA targeting. Our data demonstrate that: (*i*) HF‐diet induced hepatic steatosis is attenuated by exercise; (*ii*) Hepatic miR‐212 is upregulated in HF mice, while restored by exercise; (*iii*) miR‐212 is induced during lipogenesis in FFA‐treated HepG2 cells; (*iv*) miR‐212 upregulation induces, while miR‐212 downregulation reduces lipogenesis in HepG2 cells regardless of FFA treatment and (*v*) miR‐212 contributes to lipogenesis *via* targeting FGF‐21.

Our results demonstrating that exercise is beneficial to reduce liver steatosis and hepatic injury in HF‐diet fed mice are in line with other publications. Exercise has previously been shown to reduce hepatic lipid content *via* downregulation of lipid metabolism‐associated genes, such as FAS, SREBP‐1c, ACC and SIRT‐1 34, 35, which can be attributable to AMP‐activated protein kinase (AMPK) activation 36. Also, the effect of exercise on hepatic lipid accumulation is linked to reduced oxidative stress and improved insulin sensitivity 37, 38. Moreover, the benefit of exercise has been found in NAFLD patients engaged in exercise regardless of aerobic and resistant training 9, 11. The current study shows that 16‐week running training reduces bodyweight, attenuates liver steatosis, and restores ALT, AST, TCH and TG serum levels in HF mice, although it has previously been indicated that the beneficial effect of exercise in NAFLD patients could be independent of bodyweight loss 11, 39, 40. Thus, other mechanisms should relate exercise and reduced steatosis in NAFLD.

It has increasingly been reported that miRNA dysregulation is responsible for the development of NAFLD 14, 17. Hereby using microarray analysis, we detected a spectrum of upregulated miRNA in livers of HF mice, including miR‐34a, ‐149, ‐155, ‐205, ‐212 and ‐1224, among which miR‐34a and miR‐155 have already been reported to be dysregulated in NAFLD 25, 41, 42, 43. Furthermore, we found that HF‐diet induced upregulation of these miRNA was notably reversed by exercise. As miR‐212 was one of the most upregulated miRNA in fatty liver in the present study and FGF‐21, a central regulator for lipid metabolism, is a previously validated target gene of miR‐212, the following work was focused on the role of miR‐212 in liver steatosis.

The HF‐diet induced miR‐212 overexpression and exercise‐associated reverse of miR‐212 level were confirmed by qRT‐PCR. Then we used HepG2 cell model with or without FFA treatment which aimed to further clarify the functional and molecular mechanisms of miR‐212 in lipogenesis. Our data show that FFA‐treated HepG2 cells have increased intracellular lipid content with an elevated level of miR‐212. Furthermore, miR‐212 mimics increases, while miR‐212 inhibitor reduces lipid synthesis in HepG2 cells regardless of FFA treatment. Our findings suggest that miR‐212 upregulation is not only an accompanying phenomenon during lipogenesis, but also could be an essential molecular factor initiating lipid synthesis. In fact, miR‐212 dysregulation has previously been reported in cancers 44, 45, 46, 47, 48, 49, angiogenic responses 50, and cardiac hypertrophy 51. To our knowledge, we are the first to reveal the potential role of miR‐212 in hepatic lipogenesis which could be used as a therapeutic tool for NAFLD.

Fibroblast growth factor‐21, a potential metabolic regulator, has been shown to be protective against obesity, type 2 diabetes and hepatic steatosis in animal studies 52, 53, 54, 55. These beneficial effects of FGF‐21 are mediated by its pleiotropic metabolic actions, including increasing energy expenditure, lowering serum and hepatic lipids (*e.g*. TG and cholesterol), and increasing insulin resistance 56, 57, 58, 59, 60, 61. As FGF‐21 is a validated target gene of miR‐212 as referred to miRWalk database, we wished to determine whether miR‐212 exerts its effect on lipogenesis through targeting FGF‐21. We have found that miR‐212 negatively regulates FGF‐21 at protein but not mRNA level in HepG2 cells regardless of FFA treatment. Meanwhile, FFA treatment reduces FGF‐21 expression at both mRNA and protein levels. Furthermore, interfering FGF‐21 could abolish the lipogenesis‐reducing effect of miR‐212 inhibitor in HepG2 cells. These results clearly indicate that FGF‐21 is a target gene of miR‐212 involved in lipogenesis.

High level of circulating FGF‐21 as well as elevated hepatic FGF‐21 mRNA expression have been reported in obesity, type 2 diabetes and NAFLD in both animal models and human subjects 52, 53, 54, 55, 62, 63, 64, 65. However, it has been reported that lower level of FGF‐21 may occur when hepatic steatosis progresses to liver inflammation and/or fibrosis 65. Thus, our data showing lower FGF‐21 protein level in HF‐diet fed mice might be probably associated with more hepatic injury. In contrast, reversed FGF‐21 protein level was detected in exercised HF mice in comparison to sedentary HF mice. In fact, increased serum and liver FGF‐21 level has been reported with exercise training 66, 67. Based on current understanding of favourable metabolic effect of FGF‐21, exercise‐induced FGF‐21 might contribute to protect against hepatic steatosis and hepatic injury. Last but not least, to fully prove miR‐212 downregulation contributes to the protective effect of exercise against non‐alcoholic fatty liver *via* targeting FGF‐21, further study is required to subject miR‐212 transgenic mice to exercise training with HF diet. Taken together, this study shows that miR‐212 downregulation contributes to the protective effect of exercise against NAFLD *via* targeting FGF‐21, linking the benefit of exercise and the downregulation of miR‐212 in preventing NAFLD. Pharmacological inhibition of miR‐212 using antagomiRs, chemically engineered oligonucleotides which used to silence endogenous miRNAs, might be a novel therapeutic strategy mimicking the benefit of exercise in the treatment of NAFLD.

## Conflicts of interest

The authors declare that there are no conflicts of interest.
